# Complications and outcomes following injection of foreign material into the male external genitalia for augmentation: a single-centre experience and systematic review

**DOI:** 10.1038/s41443-023-00675-8

**Published:** 2023-03-01

**Authors:** Karl H. Pang, Karen Randhawa, Stanley Tang, Giuseppe Fallara, Athos Katelaris, Fabio Castiglione, Kamran Ahmed, Gideon Blecher, Nim Christopher, David J. Ralph, Asif Muneer, Hussain M. Alnajjar

**Affiliations:** 1 Department of Urology and Institute of Andrology, University College London Hospitals NHS Foundation Trust, London, UK; 2 Division of Surgery and Interventional Science, University College London, London, UK; 3 Department of Urology, Western Health and Social Care Trust, Ireland, UK; 4 Department of Urology, King’s College London Hospital NHS Foundation Trust, London, UK; 5 Department of Urology, St George Hospital, Sydney, NSW Australia; 6 Department of Surgery, Monash University, Melbourne, VIC Australia; 7 NIHR Biomedical Research Centre, University College London Hospitals NHS Foundation Trust, London, UK; 8 Department of Surgical Biotechnology, University College London, London, UK

**Keywords:** Surgery, Sexual dysfunction

## Abstract

Injection of exogenous material into the penis and scrotum has been performed for augmentation purposes. Complications include cosmetic dissatisfaction, penile necrosis and lymphoedema. We report the complications and outcomes from a single centre with an updated systematic review of the literature. A retrospective review of all cases presenting with foreign substance injection into the genitalia, over a 10-year period was performed. Thirty-five patients with a mean (standard deviation (SD); range) age of 36.9 (±9.1; 22–61) years at presentation were included. The mean (SD; range) time between injection and presentation was 7.8 (±5.8; 1 day–20 years) years. The most common injected substance was silicone (*n* = 16, 45.7%) and liquid paraffin (*n* = 8, 22.9%). The penile shaft (94.3%) was the most injected site. The most common presentations were cosmetic dissatisfaction (57.1%) and pain and/or swelling (45.7%). Surgery was required in 32 (91.4%) cases. Primary procedures included local excision and primary closure (*n* = 19, 59.4%), circumcision (*n* = 5, 15.6%), excision with a split skin graft or a scrotal flap reconstruction (*n* = 5, 15.6%). Three (8.6%) patients presented with necrosis and required acute debridement. Overall, 18 patients had more than 1 procedure, and 8 patients required 3 or more procedures. A systematic search of the literature identified 887 articles of which 68 studies were included for analysis. The most common substance injected was paraffin (47.7%), followed by silicone (15.8%). The majority of patients (77.9%) presented with pain, swelling or penile deformity. 78.8% of the patients underwent surgical treatment, which included excision and primary closure with or without the use of skin grafts (85.1% of all procedures), the use of flaps (12.3%) and penile amputation (*n* = 2). Complications of foreign body injection into the male genitalia can be serious resulting in necrosis and autoamputation. Surgical intervention is often required to excise abnormal tissue to manage pain and improve cosmesis.

## Introduction

Genitalia augmentation involving the penis and/or scrotum has been a topical and controversial subject for many years. The definition of a “short” or a “small-sized” penis is unclear and debatable [1]. The length and/or the girth of the penis may be augmented surgically and non-surgically, and there has been a rise in non-surgical injections of products into the penis for aesthetic purposes by patients themselves, unregulated injectors, aestheticians, who may or may not be clinicians [2–4].

Many injectable products have been used for penile augmentation. Common medical substances used include hyaluronic acid [5], polylactic acid [6], polymethylmethacrylate (PMMA) microspheres [7], autologous fat [8] and liquid silicone [9]; and some non-medical materials used, commonly injected by patients themselves usually without medical advice include, mineral oil (e.g., baby, mechanical and olive oil) [10, 11], vaseline [12] and liquid paraffin [13, 14]. Complications may occur immediately following injections resulting in collection or abscess formation and sepsis, or may occur months and years later as a result of chronic sclerosing inflammation with patients presenting with pain, penile deformity, aesthetic dissatisfaction [13, 15, 16]. Various names of sclerosing inflammation after penile injection have been used interchangeably including, foreign body giant-cell granuloma, sclerosing lipogranuloma, or terms used associated with the product injected, such as paraffinoma, siliconoma and vaselinoma [13, 15].

Management depends on the timing and type of presentation and may include, debridement of necrotic tissue, primary excision of the product with primary closure, or reconstruction with skin graft and flaps [13, 15].

We previously reported a case series of 5 patients [10], here, we present the largest UK series to date reflecting our experience of managing complications following genitalia injection of foreign material for augmentation. In addition, we provide an update of the literature via a systematic review.

## Materials and methods

### Patients

Following Institutional Review Board (IRB) approval, a retrospective review of all cases presenting with foreign substance injection into the genitalia during a 10-year period between 2010 and 2019 was performed at a single United Kingdom (UK) tertiary centre. Patients were identified through out-patient clinic, operative and histopathological databases. Data collected included patient demographics, type of substance injected, injection site, time between injection and presentation, symptoms at presentation, and management of complications. The study was reported in accordance with the STROBE checklist (Supplementary Table 1) [17].

### Systematic review

The PubMed database was searched on August 13, 2022 (Supplementary Material 1). All English articles reporting on complications of genitalia injections for augmentation were included. All titles and abstracts were screened separately by two authors (KHP and ST) initially. Full-text articles of the included abstracts were further screened (KHP and ST). Any disagreements were solved by the two screeners, in cases where no agreement was made, the senior author (HMA) made the final decision. The references of the final list of included studies were also screened for eligibility.

## Results

### Patients and complications

Overall, 35 patients, mean (SD; range) age of 36.9 (±9.3; 22–61) years presented to our centre over 10 years. The mean (SD; range) follow-up was 18.8 (±25.7; 1–120) months. Demographics, site of injection, the substance injected, and clinical presentations are detailed in Table 1. Overall, 29 (82.9%) men were from Europe, of which, 14 (48.3%) were from the UK and 13 (44.8%) were from Eastern Europe. The most commonly used product was silicone (*n* = 16, 45.7%). The most common site of injection was the penis, whereby 33 (94.3%) patients were injected into this area, of which 11 (31.4%) were also injected into their scrotum. Cosmetic dissatisfaction (*n* = 20, 57.1%) commonly associated with visible lumps and penile deformity was the most frequent presentation. The second most common presentation was pain and swelling (*n* = 16, 45.7%). Necrosis at presentation was identified in three (8.8%) patients. The mean (range) time between injection and presentation to our unit was 7.8 (1 day-20 years) years.

**Table 1:** Patient demographics and clinical details.

Clinical details	*n* (%)
Age at presentation, mean (SD; range), years	36.9 (±9.3; 22–61)
Follow-up, mean (SD; range), months	18.8 (±25.7; 1–120)
Country of origin	
UK	14 (40.0)
Eastern Europe	13 (37.1)
Southeast Asia	6 (17.1)
Other Europe	2 (5.7)
Substance injected	
Silicone	16 (45.7)
Paraffin	8 (22.9)
Vaseline	4 (11.4)
Baby oil	3 (8.6)
Autologous fat	2 (5.7)
Marble	1 (2.9)
Unknown	1 (2.9)
Number of sites injected	
1	18 (51.4)
2	12 (34.3)
3	4 (11.4)
4	1 (2.9)
Injection site	
Penis shaft	33 (94.3)
Foreskin	11 (31.4)
Scrotum	11 (31.4)
Suprapubic area	2 (5.7)
Frenulum	1 (2.9)
Time from injection to presentation, mean (SD; range), years	7.8 (±5.8; 0–20)
Clinical presentation	
Cosmetic dissatisfaction	20 (57.1)
Pain/swelling	16 (45.7)
Tight foreskin/phimosis	8 (22.9)
Necrosis	3 (8.6)

### Management

Overall, 32 (91.4%) patients underwent surgery and the other 3 (8.6%) men were managed conservatively.

During the study period, 61 procedures were performed. Overall, 14 (40%) men had 1 procedure and 1 (2.9%) patient required 5 procedures in order to remove all the products and achieve cosmetic satisfaction (Table 2).

**Table 2:** Management of penile injection complications.

Management	*n* (%)
No. of procedures	
0 (conservative management)	3 (8.6)
1	14 (40.0)
2	10 (28.6)
3	6 (17.1)
4	1 (2.9)
5	1 (2.9)
Total no. procedures performed	61
Primary procedure (*n* = 32)	
Excision + primary closure^a^	19 (59.4)
Circumcision	5 (15.6)
Excision + SSG	4 (12.5)
Debridement necrotic tissue	3 (9.4)
Excision + scrotal flap	1 (3.1)
Subsequent procedures (*n* = 29)	
Excision + primary closure	19 (65.5)
Excision + SSG	3 (10.3)
Excision + scrotal flap	3 (10.3)
Circumcision	2 (6.9)
Scrotoplasty	1 (3.5)
Revision scar	1 (3.5)

*SSG* split skin graft.

^a^Two patients had a partial scrotectomy.

**Fig. 1:**
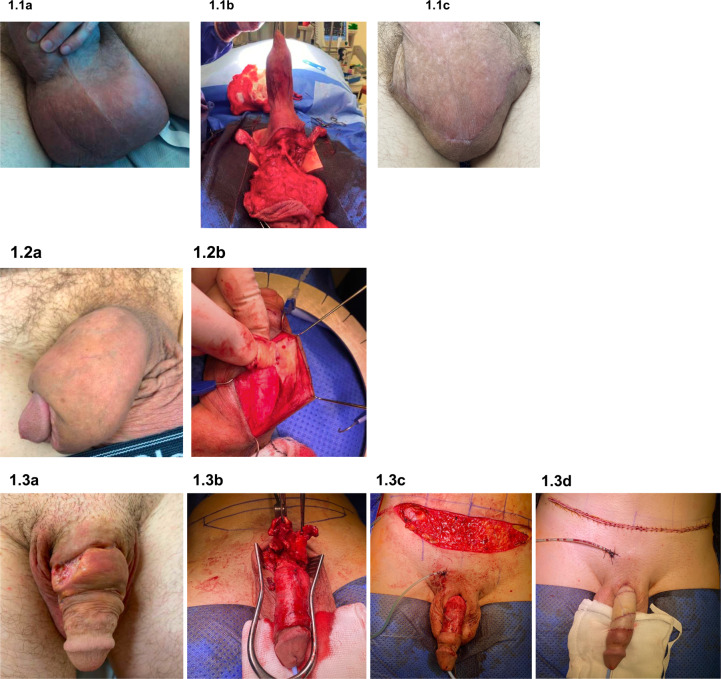
Pre-, intra-, and post-operative images of selected cases. Consent was obtained for the use of photos. **1.1a** Penoscrotal oedema secondary to silicone injection; **1.1b** excision of scrotal silicone; **1.1c** 6 weeks post-operative appearance. **1.2a** Penile oedema secondary to paraffin injection; **1.2b** dissection of foreign material. **1.3a** Penile ulceration and deformity secondary to paraffin injection; **1.3b** excision of abnormal penile tissue; **1.3c** full-thickness skin graft preparation; **1.3d** completion of excision of abnormal penile tissue and reconstruction with skin graft. Patient is now pain-free and sexually active.

The primary procedures (*n* = 32) are detailed in Table 2. A total of 19 (58.4%) patients underwent excision of the abnormal tissue and injected product with primary closure (Fig. 1). Partial scrotectomy was necessary in 5 of the 19 patients. The three patients who presented with tissue necrosis (penile, *n* = 2; scrotum, *n* = 1) underwent acute debridement. All three patients subsequently underwent deferred reconstruction with further excision of any residual product and scrotal flap coverage. Subsequent procedures (*n* = 29) included further excision of tissue and primary closure (*n* = 19, 65.5%); excision and split skin graft (*n* = 3, 10.3%); excision and scrotal flap (*n* = 3, 10.3%); scrotoplasty (*n* = 1, 3.5%) (Table 2). Out of the 61 procedures performed in our series, 38 (62.3%) were excision and closures, 7 (11.5%) procedures involved the use of a graft and 4 (6.6%) procedures required a scrotal flap.

### Histopathological findings

**Fig. 2:**
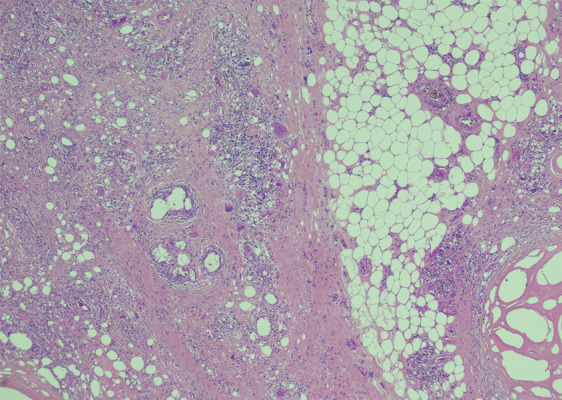
Histopathological slide demonstrating sclerosing lipogranuloma. ×4 magnification. Haematoxylin and Eosin stain. There is evidence of foreign body giant-cell reaction around lipid vacuoles and fat necrosis. Courtesy of Dr Aiman Haider, Consultant Urological Histopathologist, University College London Hospital NHS Foundation Trust. London, UK.

Within the excised tissue, histopathological findings included deposits of lipid vacuoles embedded in sclerotic stroma with associated foreign body type giant-cell granuloma. Features were in keeping with sclerosing lipogranuloma (Fig. 2). No malignancy was detected in any of the samples.

### Outcomes of systematic review

**Fig. 3:**
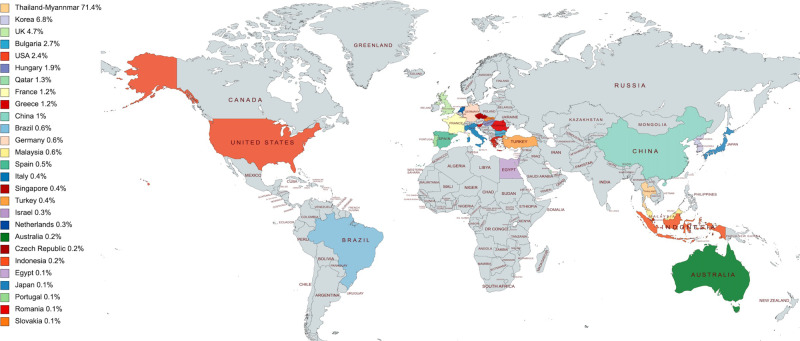
World map showing geographical distribution of reported cases.

The search retrieved 887 articles (Supplementary Fig. 1). Overall 68 studies [5, 9–12, 14, 15, 18–78] were included for analysis, which included 48 case reports (up to 2 patients), 15 case series (up to 11 patients), 1 prospective study and 5 retrospective studies (Table 3). A total of 918 men of age 17 to 77 years, with a follow-up of 1 day to 34 months were analysed. The most common substance injected was paraffin, *n* = 112 (47.7%) out of 235 patients with data, and the second commonest substance was silicone, *n* = 37 (15.8%). The time between injection and presentation was 1 day to 40 years and a majority of patients (*n* = 715, 77.9%) presented with pain, swelling or penile deformity. Phimosis or paraphimosis was reported in 15 (1.6%) men, Fournier’s gangrene occurred in 1 patient, and squamous cell carcinoma was revealed in 1 specimen. The majority (*n* = 723, 78.8%) of patients underwent surgical treatment, whilst 195 (21.2%) men were managed conservatively. Surgical treatments are summarised in Table 3, which included excision and primary closure with or without the use of skin grafts (*n* = 615, 85.1% of all procedures), the use of flaps (*n* = 89, 12.3%) and penile amputation (*n* = 2). The country with the highest number of reported complications was Thailand-Myanmar (71.4%) (Fig. 3).

**Table 3:** Summary of case reports and studies included in the current systematic review.

Author	Date	Country	Study design	No. of patients	Age (years)	Penis	Penis and scrotum	Substance		Presentation/complication		Time to presentation (year)	Surgical management		Conservative management	Follow-up (months)
Pang	2022	UK	Retrospective	35	36.9 (22–61)	24 (68.6)	11 (31.4)	Silicone	16 (45.7)	Pain	16 (45.7)	7.8 (0–20)	Excision + primary closure	19 (59.4)	3 (8.6)	18.8 (0–120)
								Paraffin	8 (22.9)	Cosmetic dissatisfaction	20 (57.0)		Circumcision	5 (15.6)		
								Vaseline	4 (11.4)	Tight foreskin/phimosis	7 (22.9)		Excision + SSG	4 (12.5)		
								Baby oil	3 (8.6)	Necrosis	2 (8.6)		Debridement necrotic tissue	3 (9.4)		
								Autologous fat	2 (5.7)				Excision + scrotal flap	1 (3.1)		
								Marble	1 (2.9)							
								Unknown	1 (2.9)							
Hrudka [18]	2022	Czech Republic	Case report	1	NR	1 (100)	0 (0)	Paraffin	1 (100)	NR	1 (100)	NR	Excision + SSG	1 (33)	0 (0)	NR
Ismy [19]	2022	Indonesia	Case report	1	42	1 (100)	0 (0)	Paraffin	1 (100)	Pain	1 (100)	5	Excision + FTSG	1 (100)	0 (0)	24
										Erectile dysfunction	1 (100)					
										Penile deformity	1 (100)					
Pereira-Loruenco [20]	2021	Spain	Case Report	1	50	1 (100)	0 (0)	Paraffin	1 (100)	Pain, cosmetic dissatisfaction	1 (100)	30	Excision + flap	1 (100)	0 (0)	3
Quan [5]	2021	China	Case series	10	30	10 (100)	0 (0)	Hyaluronic acid	10 (100)	Subcutaneous nodule	5 (50)	0–2 m	Incision and drainage	2 (20)	8 (80)	1-6
										Subcutaneous bleeding	3 (30)					
										Infection	2 (20)					
Khor [21]	2021	Australia	Case report	1	31	1 (100)	0 (0)	Hyaluronic acid	1 (100)	Pain	1 (100)	2 m		0 (0)	1 (100)	3
										Septic shock	1 (100)					
Yamasaki [22]	2021	Japan	Case report	1	65	1 (100)	0 (0)	Hyaluronic acid	1 (100)	Pain	1 (100)	NR	Partial penectomy	1 (100)	0 (0)	NR
										Necrosis	1 (100)					
Boucher [23]	2021	France	Case series	10	NR	10 (100)	0 (0)	Silicone	7 (70)	Pain	1 (100)	1.5–5	Excision + flap	9 (90)	0 (0)	12
								Paraffin	3 (30)	Painful intercourse	1 (100)		Excision + FTSG	1 (10)		
Nabiha [11]	2021	Malaysia	Case report	1	50	1 (100)	0 (0)	Olive oil	1 (100)	Painful intercourse	1 (100)	1	Excision + SSG	1 (100)	0 (0)	1
										Swelling	1 (100)					
										Penile deformity	1 (100)					
Bryce [24]	2021	USA	Case report	1	56	1 (100)	0 (0)	Hyaluronic acid	1 (100)	Pain	1 (100)	2 w		0 (0)	1 (100)	1
										Swelling	1 (100)					
Vladislav [25]	2020	Bulgaria	Case report	1	38	1 (100)	0 (0)	Paraffin	1 (100)	Pain	1 (100)	22	Excision + SSG	1 (100)	0 (0)	3 w
										Erectile dysfunction	1 (100)					
										Penile deformity	1 (100)					
Downey [15]	2019	UK	Case series	3	NR	3 (100)	0 (0)	Paraffin	3 (100)	Pain	3 (100)	NR	Excision + closure	2 (66.7)	0 (0)	NR
										Sepsis	1 (33.3)		Incision + drainage abscess	1 (33.3)		
Kim [14]	2019	Korea	Retrospective	23	45.3	21 (91)	2 (9)	Paraffin	23 (100)	Penile deformity	23 (100)	NR	Excision + closure	23 (100)	0 (0)	12.2
Dellis [26]	2018	Greece	Case series	10	20–38	7 (70)	3 (30)	Paraffin	5 (50)	Pain, cosmetic dissatisfaction	10 (100)	2	Excision + flap	10 (100)	0 (0)	2
								Silicone	3 (30)							
								Vaseline	1 (10)							
								Olive oil	1 (10)							
Furr [27]	2018	USA	Case series	11	47 (21–77)	9 (81.8)	2 (18.2)	Silicone	7 (63.6)	Pain	1	NR	Excision + closure	8 (72.7)	0 (0)	12
								Fat	1 (9.1)	Swelling	3		Excision + flap	1 (9.1)		
								Saline	1 (9.1)	Abscess	2		Debridement + SSG	1 (9.1)		
								Acellular dermis	1 (9.1)	Infection	3		Orchidectomy	1 (9.1)		
								Unknown	1 (9.1)	Nodules	1					
										Gangrene	1					
Favaret [28]	2018	Brazil	Case report	1	36	1 (100)	0 (0)	Mineral oil	1 (100)	Pain	1 (100)	1	Excision + flap	1 (100)	0 (0)	3
										Mass	1 (100)					
										Ulcer	1 (100)					
Svensoy [29]	2018	Thailand-Myanmar	Retrospective	680	32 (17–68)	Not reported		NR		Pain	571 (84)	36.7	Surgical treatment	507 (74.6)	173 (25.4)	NR
										Swelling	561 (82.5)		Circumcision	4 (0.6)		
										Induration	292 (42.9)		Excision ± graft	503 (74)		
										Purulent secretion	148 (22)					
										Ulceration	87 (12.8)					
Chon [30]	2017	Korea	Case report	1	64	1 (100)	0 (0)	Paraffin	1 (100)	Sexual dysfunction	1 (100)	35	Excision + scrotal flap	1 (100)	0 (0)	3
										Penile curvature	1 (100)					
										Voiding difficulty	1 (100)					
										Tight foreskin/phimosis	1 (100)					
Morales-Raya [31]	2017	Spain	Case report	1	42	1 (100)	0 (0)	Melted lipstick	1 (100)	Pain	1 (100)	20	Excision + closure	1 (100)	0 (0)	Lost to FU
Alcalde-Alonso [32]	2017	Spain	Case report	1	28	0 (0)	1 (100)	Paraffin	1 (100)	Thickening of scrotum/prepuce	1 (100)	8		0 (0)	Refused treatment	Lost to FU
Ahmed [10]	2017	UK	Case series	5	42.4 (28–61)	4 (80)	1 (20)	Baby oil	2 (40)	Pain	2 (40)	1 d–26 y	Debridement	1 (20)	0 (0)	NR
								Silicone	2 (40)	Swelling	2 (40)		Excision + closure	2 (40)		
								Mechanical oil	1 (20)	Voiding difficulty	1 (20)		Partial scrotectomy	1 (20)		
										Penile deformity	2 (40)		Refused surgery	1 (20)		
										Cosmetic dissatisfaction	1 (20)					
Tsili [33]	2016	Greece	Case report	1	52	1 (100)	0 (0)	Silicone	1 (100)	Swelling	1 (100)					
										Sexual dysfunction	1 (100)	10	Excision + closure	1 (100)	0 (0)	2
Singh [34]	2015	Malaysia	Case report	1	32	1 (100)	0 (0)	Paraffin	1 (100)	Pain	1 (100)	2 w	Excision + scrotal flap	1 (100)	0 (0)	3
										Swelling	1 (100)					
Cormio [35]	2014	Italy	Case report	1	35	1 (100)	0 (0)	Paraffin	1 (100)	Pain	1 (100)	7	Excision + closure	1 (100)	0 (0)	6 w
										ED	1 (100)					
										Voiding difficulty	1 (100)					
										Lump	1 (100)					
Francis [36]	2014	Singapore	Case series	4	22.5 (17–27)	4 (100)	0 (0)	Jamaica oil	4 (100)	Pain	3 (75)	6 m–4 y	Excision + scrotal flap	1 (25)	2 (50)	NR
										Swelling	3 (75)		Debridement	1 (25)		
										Hardening	1 (25)					
Majedah [37]	2014	Malaysia	Case report	1	36	1 (100)	0 (0)	Paraffin	1 (100)	Swelling	1 (100)	4	Excision + closure	1 (100)	0 (0)	NR
										Voiding difficulty	1 (100)					
Gomez-Armayones [38]	2014	Spain	Case report	1	27	1 (100)	0 (0)	Unknown	1 (100)	Ulcerated lesions	1 (100)	4		0 (0)	1 (100)	Lost to FU
Kim [39]	2014	Korea	Case series	5	NR	5 (100)	0 (0)	Paraffin	5 (100)	Necrosis	5 (100)	2 m–6 y	Excision + scrotal flap	5 (100)	0 (0)	3
De Siati [40]	2013	Italy	Case report	1	27	1 (100)	0 (0)	Paraffin	1 (100)	Pain	1 (100)	5	Excision + closure	1 (100)	0 (0)	12
										Voiding difficulty	1 (100)					
Shin [41]	2013	Korea	Retrospective	34	47.4	28 (82.4)	6 (17.6)	Paraffin	30 (88.2)	Necrosis	5 (14.7)	NR	Excision + scrotal flap	34 (100)	0 (0)	Mean, 13.5
								Vaseline	4 (11.8)	Penile deformity	Most	NR				
Oanta [42]	2013	Romania	Case report	1	29	1 (100)	0	Kanamycin	1 (100)	Fistula	1 (100)	1	Excision + closure	1 (100)	0 (0)	NR
Sukop [43]	2013	Czech Republic	Case report	1	36	1 (100)	0 (0)	Silicone	1 (100)	Infection	1 (100)	NR	Excision + closure	1 (100)	0 (0)	3
Shamsodini [9]	2012	Qatar	Case series	4	40 (30–45)	4 (100)	0 (0)	Silicone	4 (100)	Infection	2 (50)	11 m (4–15)	Excision + delayed closure	2 (50)	0 (0)	NR
										Painful intercourse	2 (50)		Excision + flap	2 (50)		
Sejben [44]	2012	Hungary	Case report	1	34	1 (100)	0 (0)	Petroleum jelly	1 (100)	Pain	1 (100)	3	Excision	1 (100)	0 (0)	NR
										Penile deformity	1 (100)					
										Inguinal lymphadenopathy	1 (100)					
Bayraktar [45]	2012	Turkey	Case report	2	22.5 (19–22)	2 (100)		Paraffin	2 (100)	Pain	2 (100)	5–6 d	Excision + closure	2 (100)	0 (0)	24
										Penile deformity	2 (100)					
Oñate Celdran [46]	2012	Spain	Case report	1	32	1 (100)	0 (0)	Paraffin	1 (100)	Pain	1 (100)	2	Excision + closure	1 (100)	0 (0)	NR
										Swelling	1 (100)					
Inn [47]	2012	Malaysia	Case series	3	45.7 (32–59)	3 (100)	0 (0)	Silicone	2 (66.6)	Pain	3 (100)	4.7 (4–5)	Excision + SSG	3 (100)	0 (0)	1
								Paraffin	1 (33.3)	Penile deformity	3 (100)					
										Swelling	3 (100)					
Karakan [48]	2012	Turkey	Case report	1	42	1 (100)	0 (0)	Vaseline	1 (100)	Pain	1 (100)	1 m	Excision + SSG	1 (100)	0 (0)	7 d
										Lump	1 (100)					
Kadouch [49]	2012	Netherlands	Case series	3	45 (38–54)	2 (66)	1 (34)	Polyalkylimide	1 (33)	Infection	2 (66)	2–6 m	Excision + SSG	1 (33)	1 (33)	12
								Polyacrylamide	1 (33)	Pain	1 (33)		Excision + closure	1 (33)		
								Silicone	1 (33)							
Bachmeyer [50]	2011	France	Case report	1	30	1 (100)	0 (0)	Paraffin	1 (100)	Pain	1 (100)	10	Excision + closure	1 (100)	0 (0)	Lost to FU
										Swelling	1 (100)					
Manny [51]	2011	USA	Case report	3	43.3 (39–47)	3 (100)	0 (0)	Mineral oil	3 (100)	Pain	3 (100)	2 m–4 y	Excision + SSG	1 (33.3)	0 (0)	1-3
										Swelling	1 (33.3)		Excision + scrotal flap	1 (33.3)		
										Phimosis	1 (33.3)		Excision + closure	1 (33.3)		
										Penile deformity	3 (100)					
Foxton [52]	2011	Australia	Case report	1	25	1 (100)	0 (0)	Oil	1 (100)	Ulcer	1 (100)	1.5	Awaiting	1 (100)	0 (0)	NR
										Penile deformity	1 (100)					
Bobik [53]	2011	Slovakia	Case report	1	33	1 (100)	0 (0)	Vaseline	1 (100)	Ulcers	1 (100)	10		0 (0)	1 (100)	NR
Al-Ansari [54]	2010	Qatar	Case series	8	38.6 (28–50)	8 (100)	0 (0)	Cod liver oil	8 (100)	Infection	8 (100)	2 w	Scrotal debulking	2 (25)	0 (0)	NR
													Excision + flap	5 (63)		
													Excision + closure	1 (12)		
Bjurlin [55]	2010	USA	Case report	1	35	1 (100)	0 (0)	Mineral oil	1 (100)	Penile deformity	1 (100)	1	Excision + SSG	1 (100)	0 (0)	NR
										Pain	1 (100)					
										Sexual dysfunction	1 (100)					
Ponyai [56]	2010	Hungary	Case report	1	29	1 (100)	0 (0)	Paraffin	1 (100)	Pain	1 (100)	14	Excision + SSG	1 (100)	0 (0)	NR
										Lesion	1 (100)					
Picozzi [57]	2010	Italy	Case report	1	38	1 (100)	0 (0)	Paraffin	1 (100)	Swelling	1 (100)	2 d	Excision + closure	1 (100)	0 (0)	2 d
										Necrosis	1 (100)					
										Paraphimosis	1 (100)					
Shaeer [58]	2009	Egypt	Case report	1	28	1 (100)	0	Gel	1 (100)	Cosmetic dissatisfaction	1 (100)	2	Excision + closure	1 (100)		5 w
										Painful intercourse	1 (100)					
Silberstein [59]	2008	USA	Case report	1	61	1 100)	0 (0)	Silicone	1 (100)	Infection	1 (100)	10		0 (0)	1 (100)	NR
										Swelling	1 (100)					
Pehlivanov [60]	2008	Bulgaria	Retrospective	25	28.3 (19–40)	25 (100)	0 (0)	Paraffin	23 (92)	Swelling	7 (30.4)	1 y (6 m–2 y)	Excision + SSG	4 (16)	2 (8)	NR
								Firm, non-fluid	2 (8)	Ulcer	11 (47.8)		Excision + scrotal flap	5 (20)		
										Phimosis	2 (8.7)		Excision + closure	14 (56)		
										Fistula	3 (13)					
Nyirady [12]	2008	Hungary	Prospective	16	31.6 (22–44)	16 (100)	0 (0)	Vaseline	16 (100)	Pain	9 (56.3)	1 d–2 y	Excision + scrotal flap	7 (43.7)	0 (0)	24
										Swelling	11 (68.8)		Excision + closure	9 (56.3)		
										Necrosis	5 (31.3)					
										Phimosis	6 (37.5)					
Dachlan [61]	2007	Indonesia	Case report	1	30	1 (100)	0 (0)	Silicone	1 (100)	Cosmetic dissatisfaction	1 (100)	5 w	Excision + closure	1 (100)	0 (0)	NR
Oh [62]	2007	Korea	Case report	1	72	1 (100)	0 (0)	Metallic mercury	1 (100)	Pain	1 (100)	9	Total penectomy, perineal urethrostomy	1 (100)	0 (0)	7
										Infection	1 (100)					
Lee [63]	2007	Korea	Case report	1	42	1 (100)	0 (0)	Petroleum jelly	1 (100)	Fournier’s gangrene	1 (100)	2	Debridement + flap	1 (100)	0 (0)	14
Rosenberg [64]	2007	Israel	Case series	3	31 (28–35)	3 (100)	0 (0)	Oil	3 (100)	Pain	2 (66.6)	1	Incision paraphimotic ring	1 (33.3)	2 (66.6)	2.6 d (1–4 d)
										Swelling	3 (100)					
										Paraphimosis	1 (33.3)					
										Phimosis	1 (33.3)					
Eandi [65]	2007	USA	Case report	1	71	1 (100)	0 (0)	Unknown	1 (100)	Voiding difficulty	1 (100)	40	Excision + closure	1 (100)	0 (0)	12
										Pain	1 (100)					
										Penile deformity	1 (100)					
Akkus [66]	2006	Turkey	Case report	1	42	1 (100)	0 (0)	Vaseline	1 (100)	Lesion	1 (100)	8 m		0 (0)	1 (100)	3 w
										Penile deformity	1 (100)					
										ED	1 (100)					
Cavalcanti [67]	2006	Brazil	Case series	5	22–42	5 (100)	0 (0)	Silicone	5 (100)	Cosmetic dissatisfaction	5 (100)	Mean 16 m	Excision + graft	5 (100)	0 (0)	NR
Hohaus [68]	2003	Germany	Case report	1	30	1 (100)	0 (0)	Vaseline	1 (100)	Painful intercourse	1 (100)	8	Excision + flap	1 (100)	0 (0)	NR
Choudhury [69]	2003	UK	Case report	1	50	1 (100)	0 (0)	Baby oil	1 (100)	Necrosis	1 (100)	NR	SSG	1 (100)	0 (0)	NR
										Swelling	1 (100)					
Santos [70]	2003	Portugal	Case report	1	40	1 (100)	0 (0)	Paraffin	1 (100)	Phimosis	1 (100)	8	Excision + closure	1 (100)	0 (0)	2
										Pain	1 (100)					
										Mass	1 (100)					
Cohen [71]	2002	USA	Case report	1	64	1 (100)	0 (0)	Mineral oil	1 (100)	Phimosis	1 (100)	2	Excision + closure	1 (100)	0 (0)	6
										ED	1 (100)					
										Voiding difficulty	1 (100)					
Kalsi [72]	2002	UK	Case report	1	31	1 (100)	0 (0)	Greece gun	1 (100)	ED	1 (100)	7	Excision + closure	1 (100)	0 (0)	6
										Penile deformity	1 (100)					
Steffens [73]	2000	Germany	Case series	5	31.2 (27–40)	4 (80)	1 (20)	Vaseline	5 (100)	Cosmetic dissatisfaction	2 (40)	1 m–20 y	Excision + graft	4 (80)	0 (0)	NR
										Painful intercourse	2 (40)		Scrotal debulking	1 (20)		
										Ulceration	1 (20)		Excision + closure	1 (20)		
Ciancio [74]	2000	USA	Case report	1	55	1 (100)	0 (0)	Mineral oil	1 (100)	Pain	1 (100)	35	Excision + SSG	1 (100)	0 (0)	6
										Ulcerated mass – SCC (Bx)	1 (100)					
Muraro [75]	1996	Italy	Case report	1	32	1 (100)	0 (0)	Paraffin	1 (100)	Cosmetic dissatisfaction	1 (100)	2 m	Excision + flap	1 (100)	0 (0)	34
Wassermann [76]	1995	USA	Case report	1	42	1 (100)	0 (0)	Silicone	1 (100)	Cosmetic dissatisfaction	1 (100)	14	Excision + flap	1 (100)	0 (0)	6
										Erectile dysfunction	1 (100)					
Arthaud [77]	1973	USA	Case report	1	51	0 (0)	1 (100)	Silicone	1 (100)	Pain	1 (100)	4	Excision + closure	1 (100)	0 (0)	8
May [78]	1956	USA	Case report	1	37	1 (100)	0 (0)	Paraffin	1 (100)	Pain	1 (100)	6	Excision + scrotal flap	1 (100)	0 (0)	2
										Penile deformity	1 (100)					
										Sexual dysfunction	1 (100)					

*SSG* split skin graft, *FTSG* full-thickness skin graft.

## Discussion

Here we report the complications following male genitalia injection of foreign substances for augmentation, and our experience with managing these cases. In our study period, we had 35 patients presenting with complications, and most men were young (mean age, 36.9 years) and sexually active. Visible lumps and penile deformity resulting in cosmetic dissatisfaction occurred in 57.1% of our patients, and pain and swelling were reported in 45.7%. These were the most common presentations identified in our series, and a majority of patients (77.9%) from our systematic review also presented with pain, swelling or penile deformity The most common products used for injection in our patients were silicone (45.7%) and liquid paraffin (22.9%). These two agents were also the most injected products identified in our systematic review. The timing of presentation varied and may be a number of years (up to 20 years) following injection when chronic inflammation and sclerosing lipogranuloma had developed. However, acute complications may occur resulting in necrosis which requires immediate debridement. We encountered three cases of necrosis which required acute surgery.

The ultimate aim of treatment is to manage patients’ symptoms, prevent progression and provide the best cosmetic and functional outcomes for patients. Reconstructive surgery is associated with risks that patients must be fully informed about. When skin grafts are used, depending on the patient’s risk factors (e.g., immunosuppression, smoking, diabetes), there are associated risks and complications when grafting on poorly vascularised beds.

The treatment depends on which area has been injected or involved, patients’ symptoms and the patients’ expected outcomes. For treatment selection, we recommend MRI penis/scrotum before embarking on any surgical approach. We excise abnormal or necrotic tissue and try to minimise removing any normal skin. Where there has been excessive skin excision, penile reconstruction is performed with fenestrated split-thickness skin graft or full-thickness skin graft. In severe scrotal lymphoedema, we tend to perform a scrotectomy or “Batman” scrotectomy. The latter involves a “Batman” shape incision and creation of a neo-scrotum using the lateral scrotal flaps. This technique was described in a previous publication [79]. If there is a loss of penis in severe cases from autoamputation or necrosis, a phalloplasty at a later stage can be considered following initial debridement.

In our series, the majority were managed with primary excision and closure (62.3%). Our systematic review showed that 85.1% of all procedures were excision with primary closure with or without a graft. In our series, excision with or without grafting consisted of 73.8% of all procedures.

In those who have penile and scrotal involvement, we tend to treat them separately and perform surgery in a staged approach, treating one area first and the other area at a later date. Overall, we had 11 men who were injected into their penis and scrotum and underwent staged procedures. A stepwise surgical approach, by treating the most problematic/symptomatic part first allows wound healing before moving on to other parts of the genitalia. Often wound healing can be problematic in the management of these patients and by following a stepwise approach we can reduce wound complications. In addition, treating the scrotal lymphoedema first may improve the penile lymphoedema and hence avoid further surgery.

Downey et al. described the geographical distribution of reported cases and found that the highest incidence of reported cases was in Korea (31.7%), followed by Bulgaria (19.8%) and Hungary (14.3%). Our systematic review demonstrated that the current country with the highest number of reported complications was in Thailand-Myanmar (71.4%), this was due to the fact that the largest case series being from there [29].

The largest series identified from our search consisted of 680 men managed during a 5-year period in Thailand-Myanmar [29]. Similar to our study, the majority of patients presented with pain (84%) or swelling (82.5%). Overall, 507 (74.6%) patients required surgical treatment, which included circumcision in 4, and excision with or without graft in 503 men [29]. At the time of analysis, our series on complications represents the largest in the UK, and the second largest in the world. Shin et al. also reported a series size similar to ours, consisting of 34 patients [41]. They evaluated their surgical repair outcomes during a 6-year period comparing a T-type versus a “new” inverted V-type flap reconstruction technique and found that the latter was associated with lower rates of delayed wound healing (V-type 21.4% vs T-type 100%) and wound infection (V-type 7.1% vs T-type 100%) [41].

### Limitations

A limitation of our study is the retrospective design and the possibility of not identifying all patients from the search of our databases. In addition, the reported functional outcomes and patient satisfaction were inconsistently documented in case notes, therefore resulting in only small numbers with inconsistent data which precluded any meaningful analyses. With regard to the systematic review, our search terms may not have captured all relevant studies. A risk of bias assessment of the 68 included reports was not performed. Majority of the included studies were case reports and the reporting of outcomes amongst the included studies was heterogenous which precluded any statistical analyses.

## Conclusion

Unregulated genitalia injection for aesthetic purposes is becoming popular worldwide. Most products are non-prescribed and are readily available. Patients need to be made aware of the potential complications and the possibility of multiple surgeries to manage any complications. Complications may be severe including tissue necrosis and autoamputation. Referral to a specialist centre for excision of abnormal tissue and reconstruction is recommended to provide the best cosmetic outcomes for this group of young and sexually active men. Apart from penile injections, there are other non-surgical and surgical approaches to augmentation that provide an alternative option for patients. In Schifano et al.’s review, it was highlighted that a multidisciplinary approach is recommended for patients who seek medical advice for penile size concerns. This may require input from surgeons, psychiatrists and psychologists [80]. Education and awareness of this practice in addition to targeted regulation of such practices as well as prevention in public health agencies in communities where the practice appears to be more prevalent is paramount to prevent further morbidity.

## Supplementary information

Supplementary Mat 1

Supplementary Fig 1

Supplementary Table 1

## Data Availability

The datasets generated during and/or analysed during the current study are available from the corresponding author upon reasonable request.
