# Supplementary material for: Complications and outcomes following injection of foreign material into the male external genitalia for augmentation: a single-centre experience and systematic review
Source: Int J Impot Res. 2023 Mar 1;36(5):498–508. doi: 10.1038/s41443-023-00675-8 (PMC11251987; doi:10.1038/s41443-023-00675-8)
Supplement: Supplementary file 1 — Supplementary Mat 1 [file 41443_2023_675_MOESM1_ESM.docx]

**Supplementary Material 1.** Search terms used for the systematic review.

(penis OR scrotum OR genitalia) AND (Augmentation Or penile injection OR hyaluronic acid OR polylactic acid OR polymethylmethacrylate OR silicone OR autologous fact OR vaseline OR paraffin OR oil) AND (complications OR necrosis OR sclerosing lipogranuloma OR paraffinoma OR siliconoma OR vaselinoma OR reconstruction).
