# Supplementary material for: Complications and outcomes following injection of foreign material into the male external genitalia for augmentation: a single-centre experience and systematic review
Source: Int J Impot Res. 2023 Mar 1;36(5):498–508. doi: 10.1038/s41443-023-00675-8 (PMC11251987; doi:10.1038/s41443-023-00675-8)
Supplement: Supplementary file 2 — Supplementary Fig 1 [file 41443_2023_675_MOESM2_ESM.docx]

**Identification of studies via databases and registers**

Records removed *before screening*:

Duplicate records removed (n = 0)

Records identified from

Databases (n = 887)

**Identification**

Records excluded

Not relevant to research question:

(n = 819)

Records screened

(n = 887)

Reports sought for retrieval

(n = 68)

Reports not retrieved

(n =0)

**Screening**

Reports excluded:

(n =0)

Reports assessed for eligibility

(n =68)

Studies included in review

(n = 68)

**Included**

**Supplementary Figure 1.** PRISMA flow diagram for the current systematic review.

*From:*  Page MJ, McKenzie JE, Bossuyt PM, Boutron I, Hoffmann TC, Mulrow CD, et al. The PRISMA 2020 statement: an updated guideline for reporting systematic reviews. BMJ 2021;372:n71. doi: 10.1136/bmj.n71

For more information, visit: <http://www.prisma-statement.org/>
